# Diaqua­bis(5-phenyl-1*H*-pyrazole-3-carboxyl­ato)copper(II)

**DOI:** 10.1107/S1600536808000810

**Published:** 2008-01-16

**Authors:** Yuan-Hui Wu, Tian-Mo Liu, Su-Xing Luo

**Affiliations:** a School of Materials Science and Engineering, Chongqing University, Chongqing 400045, People’s Republic of China; b Department of Chemistry, Zunyi Normal College, Zunyi 563002, People’s Republic of China

## Abstract

In the centrosymmetric title compound, [Cu(C_10_H_7_N_2_O_2_)_2_(H_2_O)_2_], the Cu^II^ ion occupies an inversion centre and exhibits a distorted octa­hedral geometry. The phenyl and pyrazole rings of the ligand are twisted by an angle of 11.36 (8)°. In the crystal structure, mol­ecules are linked into a two-dimensional network parallel to the (010) plane by O—H⋯O and N—H⋯O hydrogen bonds.

## Related literature

For ligand preparation, see: Crane *et al.* (1999 ▶); Gharbaoui *et al.* (2007 ▶). For general background, see: van Herk *et al.* (2003 ▶); Knopp (1999 ▶).
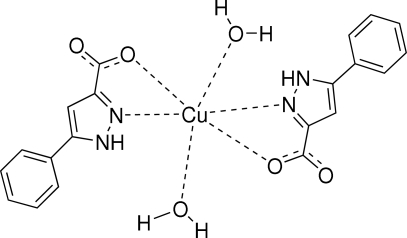


## Experimental

### 

#### Crystal data

[Cu(C_10_H_7_N_2_O_2_)_2_(H_2_O)_2_]
*M* *_r_* = 473.92
Monoclinic, 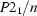

*a* = 5.0443 (6) Å
*b* = 32.161 (4) Å
*c* = 6.3234 (8) Å
β = 106.293 (1)°
*V* = 984.6 (2) Å^3^
*Z* = 2
Mo *K*α radiation
μ = 1.16 mm^−1^
*T* = 292 (2) K
0.35 × 0.25 × 0.17 mm

#### Data collection

Bruker SMART CCD area-detector diffractometer
Absorption correction: multi-scan (*SADABS*; Sheldrick, 1996 ▶) *T*_min_ = 0.690, *T*_max_ = 0.829
8611 measured reflections
2254 independent reflections
1907 reflections with *I* > 2σ(*I*)
*R*_int_ = 0.027

#### Refinement

*R*[*F* ^2^ > 2σ(*F* ^2^)] = 0.037
*wR*(*F* ^2^) = 0.084
*S* = 1.08
2254 reflections
150 parameters
3 restraints
H atoms treated by a mixture of independent and constrained refinement
Δρ_max_ = 0.34 e Å^−3^
Δρ_min_ = −0.32 e Å^−3^

### 

Data collection: *SMART* (Bruker, 1997 ▶); cell refinement: *SAINT* (Bruker, 1997 ▶); data reduction: *SAINT*; program(s) used to solve structure: *SHELXS97* (Sheldrick, 2008 ▶); program(s) used to refine structure: *SHELXL97* (Sheldrick, 2008 ▶); molecular graphics: *SHELXTL* (Sheldrick, 2008 ▶); software used to prepare material for publication: *SHELXL97*.

## Supplementary Material

Crystal structure: contains datablocks global, I. DOI: 10.1107/S1600536808000810/ci2551sup1.cif

Structure factors: contains datablocks I. DOI: 10.1107/S1600536808000810/ci2551Isup2.hkl

Additional supplementary materials:  crystallographic information; 3D view; checkCIF report

## Figures and Tables

**Table:** 

Cu1—N1	1.9572 (17)
Cu1—O1	1.9968 (14)
Cu1—O3	2.5400 (19)

**Table:** 

N1—Cu1—N1^i^	180
N1—Cu1—O1^i^	98.56 (6)
N1—Cu1—O1	81.44 (6)
O1^i^—Cu1—O1	180
N1—Cu1—O3	87.85 (7)
N1^i^—Cu1—O3	92.15 (7)
O1^i^—Cu1—O3	91.56 (6)
O1—Cu1—O3	88.44 (6)

Symmetry code: (i) 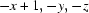.

**Table 2:** Hydrogen-bond geometry (Å, °)

*D*—H⋯*A*	*D*—H	H⋯*A*	*D*⋯*A*	*D*—H⋯*A*
O3—H1*W*⋯O2^ii^	0.83 (3)	1.88 (3)	2.679 (3)	161 (3)
N2—H2⋯O3^iii^	0.86	1.93	2.719 (3)	152
O3—H2*W*⋯O1^iv^	0.83 (3)	2.04 (3)	2.773 (3)	149 (3)

Symmetry codes: (ii) 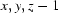; (iii) 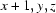; (iv) 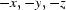.

**Fig. 1.**
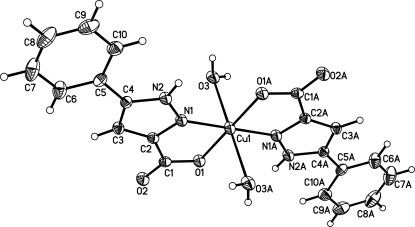
The molecular structure of the title compound, showing 30% probability displacement ellipsoids and the atomic numbering. Atoms labelled with the suffix A are generated by the symmetry operation (-x + 1, -y, -z).

## supplementary crystallographic information

### Comment

Nicotinic acid as a hypolipidemic agent appears to have good potential to increase HDL cholesterol levels to a greater extent (Knopp, 1999). However, it has severe skin flushing side effect. In the search for novel agonists for nicotinic acid receptor, substituted pyrazole-3-carboxylic acids were found have substantial affinity for cloned G protein-coupled nicotinic acid receptor (van Herk *et al.*, 2003). We report here the crystal structure of the title Cu^II^ complex with 5-phenyl-1*H*-pyrazole-3-carboxylic acid.

The asymmetric unit contains one-half of a formula unit (Fig. 1). The Cu^II^ ion occupies an inversion centre and exhibits a distorted octahedral geometry. The phenyl (C5—C10) and pyrazole (N1/N2/C2/C3/C4) rings form a dihedral angle of 11.36 (8)°. The dihedral angle between the Cu1/O1/C1/C2/N1 and N1/N2/C2/C3/C4 planes is 3.8 (1)°.

The molecules are linked into a two-dimensional network parallel to the (0 1 0) plane by O—H···O and N—H···O hydrogen bonds (Table 2).

### Experimental

5-Phenyl-1*H*-pyrazole-3-carboxylic acid was synthesized according to the reported procedure (Gharbaoui *et al.*, 2007;Crane *et al.*, 1999). 5-Phenyl-1*H*-pyrazole-3-carboxylic acid (1.0 g, 5.3 mmol) and Cu(OAc)_2_.2H_2_O (0.75 g, 2.7 mmol) were heated in H_2_O (200 ml) for 4 h with stirring. The resulting precipitate was filtered off to obtain the title compound (1.0 g, 80%). Single crystals suitable for X-ray diffraction were obtained by recrystallization from dimethylformamide-water (1:1 *v*/*v*) solution.

### Refinement

The water H atoms were located and isotropically refined, with the O—H and H···H distances restrained to 0.84 (1) and 1.37 (2) Å, respectively. The remaining H atoms were positioned geometrically (N—H = 0.86 Å and C—H = 0.93 Å) and constrained to ride on their parent atoms, with *U*_iso_(H) = 1.2*U*_eq_(C).

### Crystal data

**Table:**

[Cu(C_10_H_7_N_2_O_2_)_2_(H_2_O)_2_]	*F*_000_ = 486
*M**_r_* = 473.92	*D*_x_ = 1.599 Mg m^−^^3^
Monoclinic, *P*2_1_/*n*	Mo *K*α radiation λ = 0.71073 Å
Hall symbol: -P 2yn	Cell parameters from 2772 reflections
*a* = 5.0443 (6) Å	θ = 2.5–26.6º
*b* = 32.161 (4) Å	µ = 1.16 mm^−^^1^
*c* = 6.3234 (8) Å	*T* = 292 (2) K
β = 106.293 (1)º	Block, blue
*V* = 984.6 (2) Å^3^	0.35 × 0.25 × 0.17 mm
*Z* = 2	

### Data collection

**Table:**

Bruker SMART CCD area-detector diffractometer	2254 independent reflections
Radiation source: fine-focus sealed tube	1907 reflections with *I* > 2σ(*I*)
Monochromator: graphite	*R*_int_ = 0.027
*T* = 292(2) K	θ_max_ = 27.5º
φ and ω scans	θ_min_ = 2.5º
Absorption correction: multi-scan(SADABS; Sheldrick, 1996)	*h* = −6→6
*T*_min_ = 0.690, *T*_max_ = 0.829	*k* = −41→39
8611 measured reflections	*l* = −8→8

### Refinement

**Table:**

Refinement on *F*^2^	Secondary atom site location: difference Fourier map
Least-squares matrix: full	Hydrogen site location: inferred from neighbouring sites
*R*[*F*^2^ > 2σ(*F*^2^)] = 0.037	H atoms treated by a mixture of independent and constrained refinement
*wR*(*F*^2^) = 0.084	*w* = 1/[σ^2^(*F*_o_^2^) + (0.0302*P*)^2^ + 0.72*P*] where *P* = (*F*_o_^2^ + 2*F*_c_^2^)/3
*S* = 1.08	(Δ/σ)_max_ = 0.001
2254 reflections	Δρ_max_ = 0.34 e Å^−^^3^
150 parameters	Δρ_min_ = −0.32 e Å^−^^3^
3 restraints	Extinction correction: none
Primary atom site location: structure-invariant direct methods	

### Special details

**Table:**

Geometry. All e.s.d.'s (except the e.s.d. in the dihedral angle between two l.s. planes) are estimated using the full covariance matrix. The cell e.s.d.'s are taken into account individually in the estimation of e.s.d.'s in distances, angles and torsion angles; correlations between e.s.d.'s in cell parameters are only used when they are defined by crystal symmetry. An approximate (isotropic) treatment of cell e.s.d.'s is used for estimating e.s.d.'s involving l.s. planes.
Refinement. Refinement of *F*^2^ against ALL reflections. The weighted *R*-factor *wR* and goodness of fit *S* are based on *F*^2^, conventional *R*-factors *R* are based on *F*, with *F* set to zero for negative *F*^2^. The threshold expression of *F*^2^ > σ(*F*^2^) is used only for calculating *R*-factors(gt) *etc*. and is not relevant to the choice of reflections for refinement. *R*-factors based on *F*^2^ are statistically about twice as large as those based on *F*, and *R*- factors based on ALL data will be even larger.

### Fractional atomic coordinates and isotropic or equivalent isotropic displacement parameters (Å^2^)

**Table:**

	*x*	*y*	*z*	*U*_iso_*/*U*_eq_	
Cu1	0.5000	0.0000	0.0000	0.03166 (13)	
O1	0.3462 (3)	0.01796 (5)	0.2440 (2)	0.0325 (3)	
O2	0.3301 (4)	0.07235 (5)	0.4604 (3)	0.0404 (4)	
O3	0.0850 (4)	0.03336 (6)	−0.2706 (3)	0.0380 (4)	
N1	0.6670 (4)	0.05536 (5)	0.0513 (3)	0.0294 (4)	
N2	0.8212 (4)	0.08034 (5)	−0.0348 (3)	0.0301 (4)	
H2	0.8956	0.0730	−0.1359	0.036*	
C1	0.4084 (4)	0.05525 (7)	0.3144 (3)	0.0288 (5)	
C2	0.5895 (4)	0.07783 (7)	0.2015 (3)	0.0277 (4)	
C3	0.6964 (4)	0.11779 (7)	0.2111 (4)	0.0302 (5)	
H3	0.6732	0.1395	0.3017	0.036*	
C4	0.8454 (4)	0.11852 (6)	0.0569 (4)	0.0282 (4)	
C5	1.0091 (5)	0.15138 (7)	−0.0085 (4)	0.0328 (5)	
C6	1.0740 (6)	0.18727 (8)	0.1155 (5)	0.0473 (6)	
H6	1.0067	0.1911	0.2368	0.057*	
C7	1.2389 (7)	0.21770 (9)	0.0605 (6)	0.0641 (9)	
H7	1.2821	0.2417	0.1454	0.077*	
C8	1.3376 (6)	0.21240 (9)	−0.1183 (6)	0.0642 (9)	
H8	1.4482	0.2328	−0.1543	0.077*	
C9	1.2744 (6)	0.17720 (10)	−0.2448 (5)	0.0565 (8)	
H9	1.3419	0.1738	−0.3663	0.068*	
C10	1.1097 (5)	0.14664 (8)	−0.1918 (4)	0.0438 (6)	
H10	1.0662	0.1229	−0.2786	0.053*	
H1W	0.138 (6)	0.0417 (9)	−0.376 (4)	0.069 (10)*	
H2W	−0.041 (5)	0.0163 (8)	−0.312 (5)	0.075 (11)*	

### Atomic displacement parameters (Å^2^)

**Table:**

	*U*^11^	*U*^22^	*U*^33^	*U*^12^	*U*^13^	*U*^23^
Cu1	0.0386 (2)	0.0297 (2)	0.0341 (2)	−0.00911 (17)	0.02227 (17)	−0.00583 (17)
O1	0.0365 (9)	0.0338 (8)	0.0339 (8)	−0.0071 (7)	0.0207 (7)	−0.0019 (7)
O2	0.0523 (11)	0.0407 (9)	0.0388 (9)	−0.0031 (8)	0.0301 (8)	−0.0036 (7)
O3	0.0416 (10)	0.0443 (10)	0.0361 (9)	−0.0093 (8)	0.0239 (8)	−0.0051 (8)
N1	0.0332 (10)	0.0304 (10)	0.0301 (9)	−0.0060 (7)	0.0177 (8)	−0.0028 (7)
N2	0.0334 (10)	0.0325 (10)	0.0311 (10)	−0.0064 (8)	0.0203 (8)	−0.0029 (8)
C1	0.0276 (11)	0.0349 (12)	0.0264 (11)	0.0006 (9)	0.0115 (9)	0.0032 (9)
C2	0.0286 (11)	0.0311 (11)	0.0260 (10)	0.0004 (8)	0.0119 (9)	−0.0003 (8)
C3	0.0315 (11)	0.0298 (11)	0.0320 (12)	0.0002 (9)	0.0133 (9)	−0.0033 (9)
C4	0.0282 (10)	0.0277 (11)	0.0296 (11)	0.0005 (8)	0.0094 (9)	0.0016 (9)
C5	0.0285 (11)	0.0303 (11)	0.0402 (12)	0.0008 (9)	0.0108 (9)	0.0071 (10)
C6	0.0478 (15)	0.0366 (14)	0.0641 (18)	−0.0063 (11)	0.0264 (13)	−0.0051 (12)
C7	0.0610 (19)	0.0357 (15)	0.104 (3)	−0.0135 (13)	0.0363 (19)	−0.0046 (15)
C8	0.0521 (18)	0.0476 (17)	0.101 (3)	−0.0060 (14)	0.0345 (18)	0.0248 (17)
C9	0.0530 (17)	0.0655 (19)	0.0588 (18)	−0.0018 (14)	0.0287 (14)	0.0232 (15)
C10	0.0481 (15)	0.0462 (15)	0.0411 (14)	−0.0060 (11)	0.0189 (12)	0.0055 (11)

### Geometric parameters (Å, °)

**Table:**

Cu1—N1	1.9572 (17)	C3—C4	1.388 (3)
Cu1—N1^i^	1.9573 (17)	C3—H3	0.93
Cu1—O1^i^	1.9968 (14)	C4—C5	1.470 (3)
Cu1—O1	1.9968 (14)	C5—C6	1.382 (3)
Cu1—O3	2.5400 (19)	C5—C10	1.398 (3)
O1—C1	1.287 (3)	C6—C7	1.390 (4)
O2—C1	1.231 (3)	C6—H6	0.93
O3—H1W	0.827 (10)	C7—C8	1.368 (5)
O3—H2W	0.825 (10)	C7—H7	0.93
N1—C2	1.336 (3)	C8—C9	1.371 (5)
N1—N2	1.336 (2)	C8—H8	0.93
N2—C4	1.349 (3)	C9—C10	1.387 (3)
N2—H2	0.86	C9—H9	0.93
C1—C2	1.496 (3)	C10—H10	0.93
C2—C3	1.388 (3)		
			
N1—Cu1—N1^i^	180	C3—C2—C1	135.23 (19)
N1—Cu1—O1^i^	98.56 (6)	C4—C3—C2	105.36 (19)
N1^i^—Cu1—O1^i^	81.44 (6)	C4—C3—H3	127.3
N1—Cu1—O1	81.44 (6)	C2—C3—H3	127.3
N1^i^—Cu1—O1	98.56 (6)	N2—C4—C3	106.56 (18)
O1^i^—Cu1—O1	180	N2—C4—C5	121.60 (19)
N1—Cu1—O3	87.85 (7)	C3—C4—C5	131.8 (2)
N1^i^—Cu1—O3	92.15 (7)	C6—C5—C10	118.6 (2)
O1^i^—Cu1—O3	91.56 (6)	C6—C5—C4	120.2 (2)
O1—Cu1—O3	88.44 (6)	C10—C5—C4	121.2 (2)
C1—O1—Cu1	115.29 (13)	C5—C6—C7	120.6 (3)
Cu1—O3—H1W	107 (2)	C5—C6—H6	119.7
Cu1—O3—H2W	110 (2)	C7—C6—H6	119.7
H1W—O3—H2W	111 (2)	C8—C7—C6	120.1 (3)
C2—N1—N2	106.51 (17)	C8—C7—H7	120.0
C2—N1—Cu1	114.33 (14)	C6—C7—H7	120.0
N2—N1—Cu1	138.77 (14)	C7—C8—C9	120.4 (3)
N1—N2—C4	111.41 (17)	C7—C8—H8	119.8
N1—N2—H2	124.3	C9—C8—H8	119.8
C4—N2—H2	124.3	C8—C9—C10	120.1 (3)
O2—C1—O1	125.27 (19)	C8—C9—H9	120.0
O2—C1—C2	120.58 (19)	C10—C9—H9	120.0
O1—C1—C2	114.14 (18)	C9—C10—C5	120.3 (3)
N1—C2—C3	110.16 (18)	C9—C10—H10	119.9
N1—C2—C1	114.60 (18)	C5—C10—H10	119.9
			
N1—Cu1—O1—C1	2.75 (15)	O1—C1—C2—C3	176.1 (2)
N1^i^—Cu1—O1—C1	−177.25 (15)	N1—C2—C3—C4	0.0 (2)
O3—Cu1—O1—C1	−85.31 (15)	C1—C2—C3—C4	−178.6 (2)
O1^i^—Cu1—N1—C2	175.94 (15)	N1—N2—C4—C3	−0.1 (2)
O1—Cu1—N1—C2	−4.06 (15)	N1—N2—C4—C5	179.06 (19)
O3—Cu1—N1—C2	84.69 (16)	C2—C3—C4—N2	0.1 (2)
O1^i^—Cu1—N1—N2	4.4 (2)	C2—C3—C4—C5	−179.0 (2)
O1—Cu1—N1—N2	−175.6 (2)	N2—C4—C5—C6	−167.6 (2)
O3—Cu1—N1—N2	−86.8 (2)	C3—C4—C5—C6	11.3 (4)
C2—N1—N2—C4	0.1 (2)	N2—C4—C5—C10	10.3 (3)
Cu1—N1—N2—C4	172.02 (17)	C3—C4—C5—C10	−170.8 (2)
Cu1—O1—C1—O2	178.61 (18)	C10—C5—C6—C7	−0.8 (4)
Cu1—O1—C1—C2	−1.0 (2)	C4—C5—C6—C7	177.2 (3)
N2—N1—C2—C3	0.0 (2)	C5—C6—C7—C8	0.3 (5)
Cu1—N1—C2—C3	−174.22 (15)	C6—C7—C8—C9	0.2 (5)
N2—N1—C2—C1	178.84 (17)	C7—C8—C9—C10	−0.1 (5)
Cu1—N1—C2—C1	4.7 (2)	C8—C9—C10—C5	−0.5 (4)
O2—C1—C2—N1	178.0 (2)	C6—C5—C10—C9	0.9 (4)
O1—C1—C2—N1	−2.4 (3)	C4—C5—C10—C9	−177.1 (2)
O2—C1—C2—C3	−3.5 (4)		

Symmetry codes: (i) −*x*+1, −*y*, −*z*.

### Hydrogen-bond geometry (Å, °)

**Table:**

*D*—H···*A*	*D*—H	H···*A*	*D*···*A*	*D*—H···*A*
O3—H1W···O2^ii^	0.83 (3)	1.88 (3)	2.679 (3)	161 (3)
N2—H2···O3^iii^	0.86	1.93	2.719 (3)	152
O3—H2W···O1^iv^	0.83 (3)	2.04 (3)	2.773 (3)	149 (3)

Symmetry codes: (ii) *x*, *y*, *z*−1; (iii) *x*+1, *y*, *z*; (iv) −*x*, −*y*, −*z*.
